# Inhibition of Inflammation and iNOS Improves Lymphatic Function in Obesity

**DOI:** 10.1038/srep19817

**Published:** 2016-01-22

**Authors:** Jeremy S. Torrisi, Geoffrey E. Hespe, Daniel A. Cuzzone, Ira L. Savetsky, Matthew D. Nitti, Jason C. Gardenier, Gabriela D. García Nores, Dawit Jowhar, Raghu P. Kataru, Babak J. Mehrara

**Affiliations:** 1 The Department of Surgery, Division of Plastic and Reconstructive Surgery, Memorial Sloan Kettering Cancer Center, New York, NY

## Abstract

Although recent studies have shown that obesity decreases lymphatic function, the cellular mechanisms regulating this response remain unknown. In the current study, we show that obesity results in perilymphatic accumulation of inflammatory cells and that local inhibition of this response with topical tacrolimus, an inhibitor of T cell differentiation, increases lymphatic vessel density, decreases perilymphatic iNOS expression, increases lymphatic vessel pumping frequency, and restores lymphatic clearance of interstitial fluid to normal levels. Although treatment of obese mice with 1400W, a selective inhibitor of iNOS, also improved lymphatic collecting vessel contractile function, it did not completely reverse lymphatic defects. Mice deficient in CD4^+^ cells fed a high fat diet also gained weight relative to controls but were protected from lymphatic dysfunction. Taken together, our findings suggest that obesity-mediated lymphatic dysfunction is regulated by perilymphatic accumulation of inflammatory cells and that T cell inflammatory responses are necessary to initiate this effect.

Recent laboratory and clinical studies have shown that obesity has significant detrimental effects on the lymphatic system. For example, hypercholesterolemic mice or mice with diet-induced obesity (DIO) have abnormal lymphatic architecture, impaired initial lymphatic uptake, decreased immune cell trafficking from the periphery to regional lymph nodes, and decreased collecting vessel contraction capacity^1^,^2^,^3^. Consistent with these findings, clinical studies have shown that obese patients have a markedly impaired ability to clear macromolecules through adipose tissue lymphatics and have an increased risk of developing lymphedema either spontaneously or after lymphatic injury^4^,^5^,^6^. Similarly, animal studies have shown that obesity exacerbates the effects of lymphatic injury by increasing tissue inflammation as well as the severity of lymphedema^7^.

A major cause of obesity-associated pathology in a variety of conditions including metabolic syndrome, tumor growth/metastasis, and endothelial dysfunction, is chronic low-grade inflammation and release of inflammatory cytokines^8^,^9^,^10^,^11^,^12^,^13^,^14^,^15^,^16^,^17^. Chronic inflammation in obesity is thought to be initiated by T cell inflammatory reactions that precede and are necessary for macrophage homing to visceral adipose tissues^11^. Subsequently, macrophage differentiation and phagocytosis of necrotic adipocytes leads to the release of inflammatory cytokines, increased inflammatory cell recruitment, and progressive inflammation. However, while it is clear that obesity-induced inflammation is a major mechanism regulating generalized pathologic responses and that obesity causes marked lymphatic abnormalities, it remains unknown whether inflammation plays a causal role in regulating obesity-mediated lymphatic dysfunction. In addition, the cellular effects of chronic obesity-induced inflammation on collecting lymphatic pumping and trafficking of immune cells remain unknown. Understanding how obesity regulates lymphatic function is important since a major role for the lymphatic system is the clearance of inflammatory responses. Based on this rationale, we and others have hypothesized that obesity-induced lymphatic dysfunction augments inflammatory responses, thereby amplifying the pathology of obesity in other organ systems^18^,^19^. This hypothesis is also supported by recent studies demonstrating that lymphatic function critically contributes to reverse cholesterol transport and that animals with abnormal lymphatics are more prone to developing atherosclerotic lesions^19^,^20^,^21^.

In the current study, we used a mouse model of diet-induced obesity to test the hypothesis that perilymphatic low-grade inflammation is a major regulator of lymphatic dysfunction and that this process is dependent on T cell infiltration, decreased capillary lymphatic density, expression of inducible nitric oxide synthase (iNOS), and inhibition of collecting lymphatic pumping frequency. To avoid confounding effects of systemic T cell depletion, we tested our hypothesis by treating lean and obese mice with topical tacrolimus, a potent anti-T cell therapeutic agent, in order to locally deplete T cells. In other experiments, we used a specific small molecule inhibitor of iNOS to determine how obesity-mediated changes in tissue iNOS expression regulate collecting lymphatic pumping frequency. We show that local T cell inhibition markedly decreases perilymphatic inflammation and restores lymphatic function in obese mice by increasing capillary lymphatic density and augmenting collecting lymphatic contraction frequency. Mice lacking CD4^+^ cells fed a high fat diet displayed no evidence of perilymphatic inflammation and had normal lymphatic function despite a modest weight gain. Finally, we found that targeted iNOS inhibition also improved collecting lymphatic contraction frequency in obese mice. However, this treatment was less effective in restoring overall lymphatic function as compared with tacrolimus, having less pronounced effects on perilymphatic inflammation and lymphatic vessel density.

## Results

### Obesity results in systemic perilymphatic inflammation

**Figure 1:**
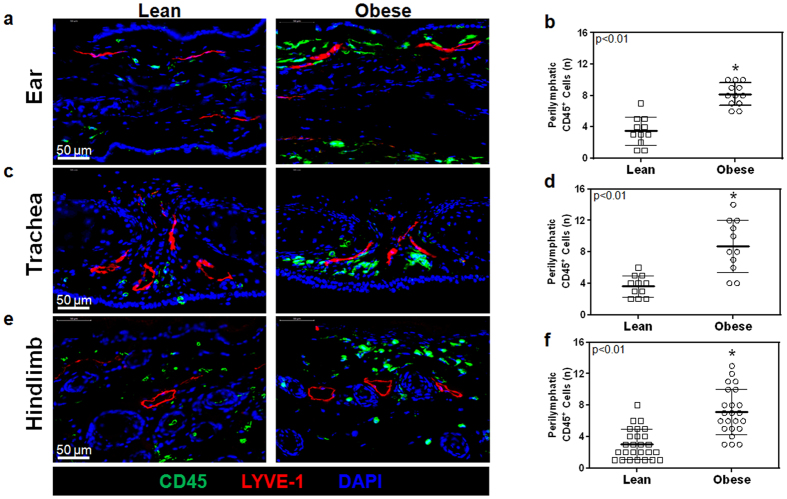
Obesity causes systemic perilymphatic inflammation. (**a**) Representative high power photomicrographs (40x) and quantification of (**b**) perilymphatic inflammation in ear sections stained for the CD45 (pan-leukocyte marker; green) and LYVE-1 (lymphatic vessels; red) (n = 5 with 4–5 HPF/animal, p < 0.01). (**c**) Representative high power photomicrographs (40x) and quantification of (**d**) perilymphatic inflammation in trachea sections stained for CD45 and LYVE-1 (n = 5 with 4–5 HPF/animal, p < 0.01). (**e**) Representative high power photomicrographs (40x) and quantification of (**f**) perilymphatic inflammation in hindlimb sections stained for CD45 and LYVE-1 (n = 5 with 4–5 HPF/animal, p < 0.01).

As expected, male C57BL/6NTac mice fed a high fat diet (HFD) for 8–10 weeks developed significant obesity and weighed an average of 56% more than their age-matched normal chow diet controls (Supplemental Fig. S1A). In addition, obese animals had significantly elevated levels of LDL, HDL, and total cholesterol as compared to lean controls (Supplemental Fig. S1B–D). Analysis of tissue sections harvested from the ear, trachea, and hindlimb demonstrated a 2–3 fold increase in the number of infiltrating CD45^+^ cells (a pan-leukocyte marker) within a 50 μm radius of tissue capillary lymphatic vessels (LYVE-1^+^), suggesting that perilymphatic inflammation in obesity is a systemic phenomenon (Fig. 1a–f).

### Topical tacrolimus decreases perilymphatic inflammation in obese mice

**Figure 2:**
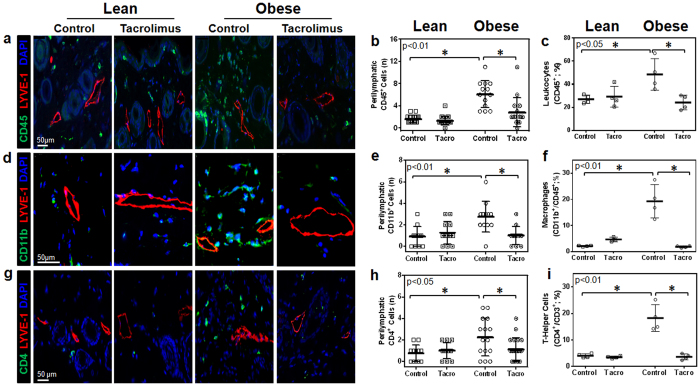
Topical tacrolimus decreases perilymphatic inflammation in obese mice. (**a**) Representative high power photomicrographs (40x) and quantification of (**b**) perilymphatic inflammation in hindlimb sections stained for CD45 (green) and LYVE-1 (red; n = 5 animals with 4–5 HPF/animal, p < 0.01). (**c**) Hindlimb tissues assessed using flow cytometry to identify CD45^+^ cells as a percentage of live cells (p < 0.05). (**d**) Representative high power photomicrographs (80x) and quantification of (**e**) perilymphatic inflammation in hindlimb sections stained for CD11b (green) and LYVE-1 (red; n = 5 animals with 4–5 HPF/animal, p < 0.01). (**f**) Hindlimb tissues assessed using flow cytometry to identify macrophages (CD11b^+^/CD45^+^) as a percentage of total live cells (n = 4 animals/group, p < 0.01). (**g**) Representative high power photomicrographs (40x) and quantification of (**h**) perilymphatic inflammation in dermal hindlimb sections stained for CD4 (green) and LYVE-1 (red; n = 5 animals with 4–5 HPF/animal, p < 0.05). (**i**) Hindlimb tissues assessed using flow cytometry to identify T helper cells (CD4^+^/CD3^+^/CD45^+^) as a percentage of total live cells (p < 0.01).

Mice treated with topical tacrolimus applied unilaterally to the hindlimb (0.05 gm twice daily) for two weeks had very low levels of systemic absorption (1–2 ng/ml in the serum) and did not display significant changes in body weight or metabolic parameters including fasting serum glucose or insulin levels (Supplemental Fig. S1A–F). However, topical application of tacrolimus markedly decreased perilymphatic accumulation of leukocytes (CD45^+^ cells), macrophages (CD11b^+^ cells), and T cells (CD4^+^) in the treated hindlimb as assessed using immunofluorescent localization and flow cytometry on single cell tissue digests (Fig. 2a–i and Supplemental Fig. S2A–C). In fact, obese mice treated with topical tacrolimus had tissue inflammatory cell content that was within range of levels observed in lean mice. We observed no significant changes in these parameters in lean mice treated with topical tacrolimus (Fig. 2a–i and Supplemental Fig. S2A–C).

### Tacrolimus restores lymphatic transport capacity and DC migration in obese mice

**Figure 3:**
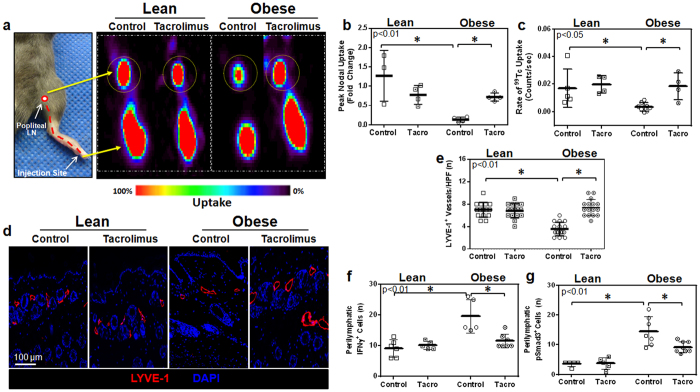
Tacrolimus restores lymphatic transport capacity in obese mice. (**a**) *Left panel:* Diagram overlay on a photograph of a distal mouse hindlimb displaying the pathway (dotted red line) of ^99m^Tc from the injection site to the popliteal lymph node. *Right panel:* Representative ^99m^Tc heat maps (red  =  highest uptake; black  =  lowest uptake) from experimental and control animals displaying uptake of ^99m^Tc to the popliteal lymph node (dotted yellow circle; n = 4 animals/group). (**b**) Quantification of peak nodal uptake of ^99m^Tc in the popliteal lymph node. Data is represented as a fold change relative to the lean control group (p < 0.01). (**c**) Quantification of the rate of uptake of ^99m^Tc to the popliteal lymph node (p < 0.05). (**d**) Representative photomicrographs (20x) and quantification of (**e**) lymphatic vessel density (LYVE-1^+^ vessels/HPF) in hindlimb sections (n = 5 animals/group with 4–5 HPF/animal, p < 0.01). (**f**) Quantification of the number of perilymphatic IFN-γ^+^ cells (p < 0.01). (**g**) Quantification of the number of perilymphatic pSMAD-3^+^ cells (p < 0.01).

A major function of the lymphatic system is to transport interstitial fluid to regional lymph nodes. To test the hypothesis that low-grade perilymphatic inflammation inhibits interstitial fluid transport, lean and obese mice were treated with either topical tacrolimus or vehicle control, which were applied unilaterally to the hindlimb for two weeks. At this time, animals were injected in the distal portion of the treated hindlimb with technetium-99 m labeled sulfur colloid (^99m^Tc; a substance cleared only by the lymphatics due to its high molecular weight) and decay-adjusted uptake of ^99m^Tc was assessed in the popliteal lymph node as previously reported^3^. As compared with lean mice, control obese animals had markedly decreased peak and rate of ^99m^Tc labeled sulfur colloid uptake by popliteal lymph nodes after peripheral hindlimb injection (Fig. 3a–c). Treatment of obese mice with tacrolimus reversed these deficits and markedly increased popliteal ^99m^Tc uptake (5.3 fold increase in peak and 15 fold increase in rate) as compared with control obese mice. These values approximated what we had observed in lean mice. Interestingly, increases in lymph node ^99m^Tc uptake after tacrolimus treatment was limited only to obese mice since we observed no changes in lymph node uptake in lean mice treated in this manner as compared to their respective controls (Fig. 3a–c). This finding suggests that the effects of tacrolimus on the lymphatic system are indirect depending on changes in inflammatory cell accumulation rather than direct effects on lymphatic endothelial cells.

Analysis of LYVE-1 vessel density in the dermis and subcutaneous tissues of vehicle treated animals demonstrated that obese mice had a significant decrease (1.9 fold) in capillary lymphatic density as compared with lean mice (Fig. 3d,e). Treatment of obese mice with topical tacrolimus normalized lymphatic vessel density (Fig. 3d,e). In contrast, similar treatment of lean mice had no effect on capillary lymphatic vessel density. Consistent with our finding of decreased inflammation after topical application of tacrolimus to the hindlimbs of obese mice, we noted a marked decrease in cells that expressed interferon-gamma (IFN-γ) and phosphorylated SMAD-3 (p-SMAD3), a downstream mediator of transforming growth factor-beta (TGF-β) signaling (Fig. 3f,g). This is important since previous studies have shown that IFN-γ^22^ and TGF-β^23^,^24^ have potent anti-lymphangiogenic activity in a number of physiologic settings, suggesting that obesity-induced reductions in lymphatic vessel density may be regulated by increased local expression of anti-lymphangiogenic cytokines.

### Tacrolimus restores dendritic cell migration and increases collecting vessel pumping frequency in obese mice

Trafficking of dendritic cells (DCs) to regional lymph nodes occurs via active and passive mechanisms within the lymphatic system and therefore is a measure of lymphatic function^25^. To test the hypothesis that topical tacrolimus improves trafficking of DCs in obese mice, we treated lean and obese mice with vehicle control or tacrolimus applied unilaterally to the hindlimb for two weeks. Eighteen hours prior to sacrifice, control and tacrolimus treated animals were injected in the distal hindlimb with DCs harvested from lean syngeneic C57BL/6 mice that express CD45.1. This approach enabled us to specifically quantify injected DCs trafficking to the popliteal lymph node since the recipient mice express CD45.2.

**Figure 4:**
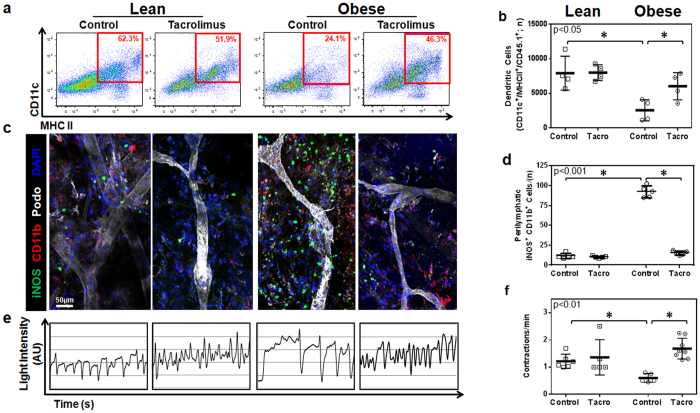
Tacrolimus restores dendritic cell migration capacity in obese mice. (**a**) Representative flow cytometry plots of CD11c^+^/MHC-II^+^/CD45.1^+^ transplanted dendritic cells collected from popliteal lymph nodes 18 hours after injection in the distal hindlimb. Red box indicates gating used to determine CD11c^+^/MHC-II^+^ double-positive dendritic cells. (**b**) Quantification of CD11c^+^/MHC-II^+^/CD45.1^+^ transplanted dendritic cells (n = 4 animals/group, p < 0.05). (**c**) Representative whole mount images of mouse ears stained for iNOS (green), CD11b (red), and Podoplanin (white). Note accumulation of iNOS^+^ cells around lymphatic collectors in obese control animals and loss of this response in obese animals treated with topical tacrolimus. (**d**) Quantification of perilymphatic iNOS^+^/CD11b^+^ cells in whole mount stained tissues (n = 4 animals/group, p < 0.001). (**e**) Representative line graphs showing lymphatic contractions over time. Peaks in intensity correspond with lymphatic contractions and are shown with arbitrary units (AU). (**f**) Quantification of lymphatic contractions (lymphatic contractions per minute; n = 5 animals per group, p < 0.01).

Consistent with our previous studies and our findings with^99m^ Tc lymph node uptake, we found that obese mice had markedly decreased trafficking of transplanted DCs (3.5x less) as compared with lean mice (Fig. 4a,b). In addition, similar to our findings in lymphoscintigraphy, we found that treatment of obese mice hindlimbs with tacrolimus markedly improved DC trafficking to regional lymph nodes and nearly completely restored this deficit to levels noted in lean controls. Also consistent with our lymphoscintigraphy findings, we noted no changes in DC trafficking in lean mice treated with topical tacrolimus, suggesting that the beneficial effects of this treatment are dependent on a local anti-inflammatory effect.

Previous studies have shown that expression of iNOS by infiltrating macrophages and monocytes impairs lymphatic transport and decreases collecting lymphatic contraction frequency^26^. Because we had found that topical tacrolimus markedly decreased perilymphatic infiltration of macrophages (CD11b^+^ cells), we next sought to test the hypothesis that topical tacrolimus improves lymphatic function in obese mice by decreasing perilymphatic inflammation and iNOS expression. In these studies, lean and obese mice were treated with vehicle or topical tacrolimus applied to the ear or hindlimb for two weeks followed by sacrifice and immunofluorescent localization of perilymphatic iNOS-expressing cells using whole mount staining (ear) or cross-sectional histology (hindlimb).

Co-localization of iNOS expression and lymphatic vessels in ear and hindlimb tissues demonstrated that obese animals had a 3–4 fold increase in the number of perilymphatic iNOS^+^ cells as compared with lean controls (Fig. 4c,d and Supplemental Fig. S3A,B). These changes could be most easily seen in whole mount stains, as this method enabled us to visualize long segments of collecting lymphatic vessels. More importantly, we found that treatment of obese mice with topical tacrolimus markedly decreased the number of perilymphatic iNOS^+^ cells (6 fold decrease in ears; nearly 2 fold decrease in hindlimbs; Fig. 4c,d and Supplemental Fig. S3A,B). These changes effectively normalized the number of iNOS^+^ cells in obese mice to levels noted in lean controls. Although previous studies have shown that iNOS can be expressed by a variety of cell types, including adventitial smooth muscle cells^27^, in our studies with obese mice we noted that iNOS was primarily expressed by inflammatory cells.

Because iNOS expression has been shown to decrease collecting lymphatic pumping frequency^26^, we next analyzed the frequency of lymphatic vessel contractions in hindlimbs of lean and obese animals treated with vehicle or topical tacrolimus using indocyanine green (ICG) lymphangiography. This analysis, consistent with previous reports^2^, demonstrated that obese vehicle treated mice have a more than 2-fold decrease in collecting lymphatic vessel contraction frequency as compared with lean controls (Fig. 4e,f and Supplemental videos S1,S2,S3–4). Treatment of obese mice with tacrolimus markedly increased collecting vessel contraction frequency to a level that was indistinguishable from lean controls. In addition, consistent with our observation that topical tacrolimus does not significantly change inflammatory cell accumulation or iNOS expression in lean mice, we noted no changes in collecting lymphatic contraction frequency in lean control mice. Taken together, our findings suggest that low grade chronic inflammatory responses in obesity result in perilymphatic inflammation and that this response impairs lymphatic transport function, at least in part, due to increased perilymphatic iNOS expression and decreased lymphatic collecting vessel contraction frequency.

### HFD fed CD4KO mice do not exhibit lymphatic dysfunction

Based on our observation that inhibition of T cell responses with topical tacrolimus markedly decreases adipose tissue inflammatory cell accumulation and improves lymphatic function, as well as previous studies demonstrating that CD4^+^ cells have profound anti-lymphangiogenic effects, we next sought to determine how loss of CD4^+^ cells modulates the effects of HFD on obesity and lymphatic dysfunction. To test this hypothesis, we fed CD4 knockout mice (CD4KO) based on a C57BL/6 background either a normal chow diet (NCD) or high fat diet (HFD) for 8–10 weeks and analyzed weight gain and lymphatic function.

**Figure 5:**
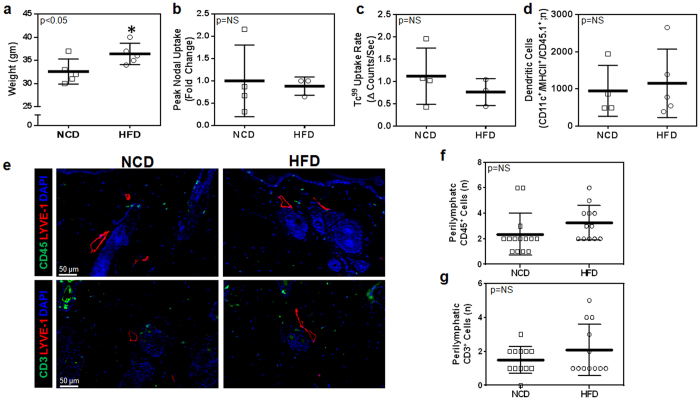
HFD fed CD4KO mice do not exhibit lymphatic dysfunction. (**a**) Weights for CD4KO mice fed either a NCD or HFD for 10 weeks (n = 5/group, p < 0.05). (**b**) Quantification of peak nodal uptake of ^99m^Tc in the popliteal lymph node. Data is represented as a fold change relative to the lean control group (n = 4 animals per group, p = NS). (**c**) Quantification of the rate of uptake of ^99m^Tc to the popliteal lymph node (p = NS). (**d**) Quantification of migration of CD11c^+^/MHC-II^+^/CD45.1^+^ transplanted dendritic cells to the popliteal lymph node (n = 4–5 animals/group, p = NS). (**e**) *Top panels:* Representative images demonstrating co-localization of CD45 (green) and LYVE-1 (red) in hindlimb tissues of NCD and HFD fed CD4KO mice. *Bottom panels:* Representative images demonstrating co-localization of CD3 (green) and LYVE-1 (red) in hindlimb tissues of NCD and HFD fed CD4KO mice. (**f**) Quantification of perilymphatic CD45^+^ cells (n = 4 animals/group; 3–4HPF/animal, p = NS). (**g**) Quantification of perilymphatic CD3^+^ cells (n = 4 animals/group; 3–4HPF/animal, p = NS).

CD4KO mice fed a HFD gained significantly more weight than control animals fed normal chow diet; however, the differences in these animals was less pronounced than those observed in wild-type mice fed either a HFD or NCD (Fig. 5a). CD4KO mice fed a HFD had no significant changes in fasting serum glucose or insulin levels but did display minor increases in total and high (but not low) density lipoprotein (Supplemental Fig. S4A–E). Interestingly, we found that CD4KO mice fed a HFD did not exhibit significant lymphatic deficits as evidenced by normal peak/rate of ^99m^Tc uptake by popliteal lymph nodes and no changes in DC trafficking as compared with control CD4KO mice fed a NCD (Fig. 5b–d). Similarly, histologic analysis of CD4KO mice fed a HFD showed that in contrast to wild-type C57BL/6 mice, this treatment does not result in perilymphatic accumulation of CD45^+^ leukocytes or CD3^+^ T cells (Fig. 5e–g). Taken together, these findings show that long term exposure to HFD, independent of obesity, does not cause lymphatic dysfunction (i.e. HFD does not have direct toxic effects on the lymphatic system). In addition, these experiments suggest that CD4^+^ cells are necessary for HFD-induced obesity.

### iNOS inhibition decreases inflammation and improves lymphatic function in obese mice

**Figure 6:**
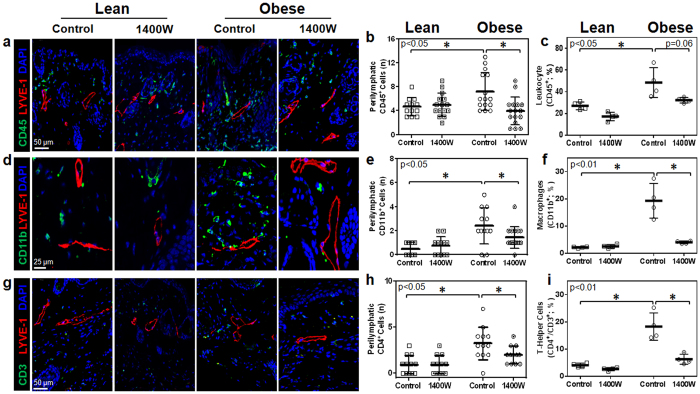
iNOS inhibition reduces inflammation in obese animals. (**a**) Representative high power photomicrographs (40x) and quantification of (**b**) perilymphatic CD45^+^ cells (green) in hindlimb sections stained for LYVE-1 (red) (n = 5 animals/group; 4–5 HPF/animal, p < 0.05). (**c**) Hindlimb tissues assessed using flow cytometry to identify CD45^+^ cells as a percentage of live cells (p < 0.05). (**d**) Representative high power photomicrographs (80x) and quantification of (**e**) perilymphatic CD11b^+^ cells (green) in hindlimb sections stained for LYVE-1 (red) (p < 0.05). (**f**) Hindlimb tissues assessed using flow cytometry to identify macrophages (CD11b^+^/CD45^+^) as a percentage of total live cells (n = 4 animals/group, p < 0.01). (**g**) Representative high power photomicrographs (40x) and quantification of (**h**) perilymphatic inflammation in dermal hindlimb sections stained for CD4 (green) and LYVE-1 (red) (n = 5, 4–5 HPF/animal, p < 0.05). (**i**) Hindlimb tissues assessed using flow cytometry to identify T helper cells (CD4^+^/CD3^+^/CD45^+^) as a percentage of total live cells (p < 0.01).

To test the hypothesis that perilymphatic iNOS expression in obese mice impairs lymphatic pumping frequency and transport function, we next analyzed the effects of a potent and specific iNOS inhibitor (1400W). Lean and obese animals were treated with 1400W (10 mg/kg/day delivered intraperitoneally) once a day for 10 days^28^,^29^,^30^, while control animals were treated with vehicle control. Treatment with 1400W was well-tolerated and resulted in no significant adverse effects, changes in weight or metabolic parameters including fasting serum glucose or insulin levels (Supplemental Fig. S5A–F). iNOS inhibition had modest, though significant, effects on inflammatory responses, decreasing perilymphatic accumulation of inflammatory cells, macrophages, and T cells in the treated hindlimb tissues (Fig. 6a–i and Supplemental Fig. S6A–C). However, this anti-inflammatory effect was generally less pronounced than what we observed in tacrolimus treated obese mice.

**Figure 7:**
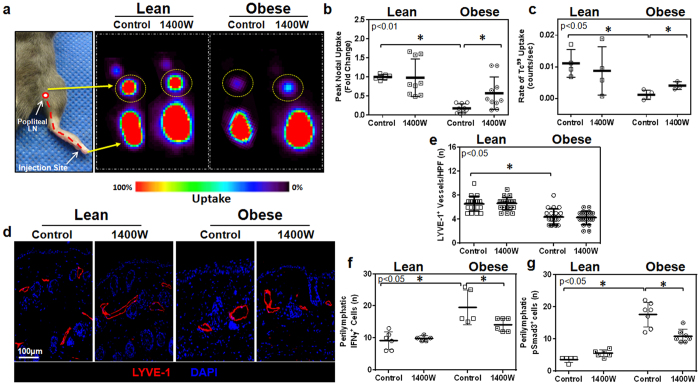
iNOS inhibition improves lymph node uptake in obese mice. (**a**) Representative ^99m^Tc heat maps from experimental animals displaying uptake of ^99m^Tc to the popliteal lymph node (yellow circles). (**b**) Quantification of peak nodal uptake of ^99m^Tc in the popliteal lymph node. Data is represented as a fold change relative to the lean control group (n = 4 animals/group, p < 0.01). (**c**) Quantification of the rate of uptake of ^99m^Tc to the popliteal lymph node (n = 4 animals/group, p < 0.05). (**d**) Representative photomicrographs (20x) and quantification of (**e**) lymphatic vessel density (LYVE-1^+^ vessels/HPF) in hindlimb sections (n = 5 animals/group with 4–5 HPF/animal, p < 0.05). (**f**) Quantification of the number of perilymphatic IFN-γ^+^ cells (p < 0.05). (**g**) Quantification of the number of perilymphatic pSMAD-3^+^ cells (p < 0.05).

As expected from our earlier findings demonstrating very few perilymphatic iNOS^+^ cells in lean mice, we found virtually no effect of 1400W treatment on ^99m^Tc uptake in lean animals (Fig. 7a–c). In contrast, treatment of obese mice with systemic 1400W significantly increased the peak (more than 3 fold), and, to a lesser though still significant extent, the rate of popliteal lymph node ^99m^Tc uptake. However, in contrast to our findings with tacrolimus, iNOS inhibition in obese mice did not normalize ^99m^Tc uptake to levels measured in lean mice. Similarly, also in contrast to our findings with tacrolimus, we found that iNOS inhibition in obese mice had very little effect on lymphatic vessel density although there were modest, though significant, decreases in the number of IFN-γ^+^ and pSMAD3^+^ cells (Fig. 7d–g).

**Figure 8:**
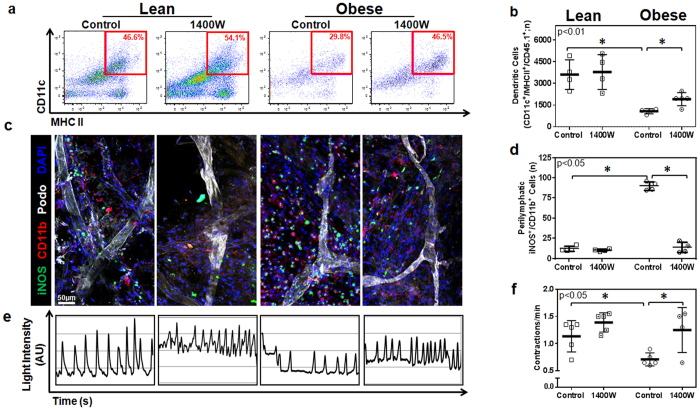
iNOS inhibition increases dendritic cell migration and increases lymphatic pumping frequency. (**a**) Representative flow cytometry plots of CD11c^+^/MHC-II^+^/CD45.1^+^ transplanted dendritic cells harvested from popliteal lymph nodes 18 hours after injection in the distal hindlimb. Red box indicates gating used to determine CD11c^+^/MHC-II^+^ double-positive dendritic cells. (**b**) Quantification of CD11c^+^/MHC-II^+^/CD45.1^+^ transplanted dendritic cells (n = 5 animals/group, p < 0.01). (**c**) Representative whole mount images of mouse ears stained for iNOS (green), CD11b (red), and Podoplanin (white). Note decreased iNOS^+^ cell accumulation in obese mice treated with 1400W. (**d**) Quantification of perilymphatic iNOS^+^/CD11b^+^ cells in whole mount stained tissues (n = 4 animals/group, p < 0.05). (**e**) Representative line graphs showing lymphatic contractions over time. Peaks in intensity correspond with lymphatic contractions and are shown as arbitrary units. (**f**) Quantification of lymphatic contractions (lymphatic contractions per minute; n = 5 animals per group, p < 0.05).

Consistent with our lymphoscintigraphic findings, we found that treatment of obese mice with systemic iNOS inhibitor modestly (though significantly) increased migration capacity of DCs as compared with control obese mice (1.8 fold increase) but, in contrast to tacrolimus, did not completely restore this defect to levels noted in lean mice (Fig. 8a,b). In addition, 1400W treatment had no discernible effect on lean mice as compared to their own respective controls. Whole mount analysis of mouse ears and immunohistochemical analysis of hindlimbs showed that iNOS inhibition significantly reduced perilymphatic iNOS^+^ cell accumulation in obese mice (more than 3 fold decrease in ears; 1.9 fold decrease in hindlimbs as compared with controls; Fig. 8c,d and Supplemental Fig. S7A, B). These findings correlated with a significant increase in lymphatic contraction frequency in 1400W-treated obese mice (1.8 fold increase vs. obese controls; Fig. 8e,f and Supplemental Videos S5,S6,S7–8). Not surprisingly, we found no differences in the number of perilymphatic iNOS^+^ cells or lymphatic contraction frequency in lean mice. Taken together, our findings with 1400W suggest that iNOS inhibition is not as effective as tacrolimus and that this decreased efficacy may be related to less pronounced improvements in lymphatic pumping frequency and lack of improvement in initial lymphatic vessel density.

## Discussion

In this study we found that inflammatory cells accumulate around lymphatic vessels, suggesting that prolonged close association of inflammatory cells with lymphatic endothelial cells (LECs) contributes to obesity-associated lymphatic dysfunction. This hypothesis is supported by the finding that tacrolimus was more effective in inhibiting subcutaneous inflammation and restoring lymphatic function as compared with the iNOS small molecule inhibitor, 1400W, which only modestly decreased perilymphatic inflammatory responses. Our hypothesis is also supported by previous studies demonstrating that perivascular inflammation is a major cause of endothelial dysfunction in obesity due to diverse mechanisms including release of reactive oxygen species, increased expression of adipokines, and impaired nitric oxide (NO) signaling pathways^16^,^31^,^32^,^33^. Taken together, our studies suggest that obesity-mediated lymphatic dysfunction can be ameliorated by local modulation of inflammatory responses and that these negative effects are mediated by multiple mechanisms, including decreased initial lymphatic vessel density as well as paracrine effects on collecting lymphatics by inflammatory cells.

We found that obese mice had decreased density of capillary lymphatics in their dermis and subcutaneous tissues and that this effect was reversed by treatment with tacrolimus but not the iNOS small molecule inhibitor, 1400W. Importantly, we did not note changes in adipose tissue architecture or volume at the site of tacrolimus application, suggesting that the increased number of lymphatic vessels resulted from capillary lymphatic proliferation rather than simply changes in the adipose tissues resulting in relative changes in lymphatic density. The fact that tacrolimus was more effective than 1400W in decreasing subcutaneous inflammation suggests that changes in lymphatic vessel density may be related to obesity-mediated chronic inflammation. In addition, because the main mechanism of action of tacrolimus is inhibition of T cell differentiation, our studies suggest that T cells play a prominent role in this response. This hypothesis is supported by our findings with CD4KO mice, as well as accumulating evidence demonstrating that T cells have potent anti-lymphangiogenic effects during inflammation and wound repair^34^,^35^. These effects result from direct effects of T cell derived cytokines and growth factors such as IFN-γ, interleukin 4, interleukin 13, and TGF-β1 in regulating LEC gene expression of lymphatic markers, cellular proliferation, migration, and tubule formation^22^,^34^,^35^,^36^,^37^. While it is also possible that tacrolimus application increased the expression of pro-lymphangiogenic growth factors such as vascular endothelial growth factor-C (VEGF-C) or VEGF-A, this effect is less likely since this treatment potently decreased the number of infiltrating macrophages, which are a major paracrine source of lymphangiogenic growth factors^38^. Indeed, recent studies have shown that obese individuals paradoxically have increased levels of tissue and serum VEGF-C expression and that blockade of VEGF-C may be an effective means of preventing insulin resistance by decreasing chemotaxis of M1 macrophages to subcutaneous adipose tissues^39^,^40^,^41^. These previous findings, together with our observation that obesity decreases lymphatic vessel density, suggest that the lymphatic vasculature in obese individuals is either less sensitive to VEGF-C stimulation, that the effects of lymphangiogenic cytokines are inhibited by other mechanisms, or both.

Previous studies have shown that T cell inflammatory responses precede and are necessary for macrophage infiltration of visceral fat in obese individuals and animals^10^,^11^,^12^,^13^,^14^. In addition, these studies have shown that inhibition of T cell responses can significantly modulate the pathological effects of obesity. For example, neutralization of CD3^+^ cells with systemically delivered antibodies markedly decreases metabolic syndrome and insulin resistance in obese mice^12^. Consistent with these results, we found that treatment of obese mice with tacrolimus, a drug that inhibits T cell proliferation and maturation by binding calcineurin and inhibiting interleukin 2 production^42^,^43^, not only decreased perilymphatic T cell infiltration but also significantly decreased overall inflammatory responses, normalized lymphatic contraction frequency, and decreased perilymphatic iNOS expression. In addition, we found that CD4KO mice are protected from obesity-induced lymphatic dysfunction. These findings are important since previous studies have shown that major hallmarks of lymphatic dysfunction in obesity and metabolic syndrome are impaired lymphatic contraction frequency and intrinsic flow generating capacity^2^,^44^. Taken together, our findings suggest that local T cell responses in obesity are necessary for perilymphatic inflammation and that this response significantly modulates collecting lymphatic vessel contractile frequency and function. In addition, because CD4KO mice did not develop lymphatic dysfunction even after prolonged HFD feeds and because local T cell inhibition did not result in significant systemic metabolic changes, our findings suggest that dietary changes alone are not sufficient to induce lymphatic dysfunction, however additional studies are needed to rule out changes in adipokines or other systemic mediators.

These findings of our current study are consistent with previous studies demonstrating that iNOS expression has regulatory effects on inflammatory cell infiltration and expression of inflammatory cytokines as a result of decreased generation of reactive oxygen species^45^. In addition, our findings of increased collecting vessel contraction frequency are consistent with previous studies demonstrating that high concentrations of NO decrease lymphatic contraction frequency and amplitude of contractions^26^,^46^,^47^,^48^. For example, in at study of rat mesenteric lymphatics, Bohlen *et al.* demonstrated that valvular and tubular lymphatic segments increase NOS expression during phasic contractions and that these changes in turn regulate lymphatic contraction and relaxation^49^. Similarly, Liao *et al.* demonstrated that basal gradients of lymphatic eNOS expression are disrupted by high levels of iNOS expression by infiltrating CD11b^+^ macrophages, resulting in decreased lymphatic contractile function and impaired immunity^50^. Other studies have shown that inflammatory cytokines known to be elevated in obesity (e.g. IL1β, TNF-α, and IL6) impair lymphatic collecting vessel contraction capacity, and, in the case of IL1β, do so by induction of iNOS expression^51^. Thus, high levels of iNOS expression by perilymphatic inflammatory cells in obese animals may impair lymphatic function by causing relaxation of lymphatic collectors thereby decreasing contraction frequency. This concept is supported by a recent study of isolated mouse collecting lymphatic vessels in which Scallan *et al.* showed that increasing levels of NO decreases contractile function and negatively influences lymph flow^52^. Additionally, changes in lymphatic preload or shear stress secondary to reductions in the number or function of initial lymphatics may contribute to decreased collecting lymphatic contraction function^50^.

In conclusion, we have shown that obesity-mediated lymphatic dysfunction is regulated by perilymphatic accumulation of inflammatory cells and that T cell inflammatory responses are necessary to initiate this effect. In addition, we have shown that inhibition of T cell infiltration locally restores lymphatic function in obese animals by increasing the density of initial lymphatics, decreasing perilymphatic iNOS expression, and increasing contraction frequency of collecting lymphatics. These findings are important since they show that obesity-induced lymphatic dysfunction is reversible and that local pharmacologic interventions are highly effective.

## Methods

### Animals

All experimental protocols were approved by the Institutional Animal Care and Use Committee at Memorial Sloan Kettering Cancer Center (MSKCC). The experimental protocols were performed in accordance with MSKCC approved guidelines. Male C57BL/6NTac mice (Taconic Biosciences, Hudson, NY) and CD4 knockout mice (CD4KO; CBY.129S2 (B6)- Cd4^tm1mak/J^; Jackson Laboratories, Bar Harbor, Maine) were kept in a light- and temperature-controlled environment. Age matched mice were fed either a normal chow diet (13% kcal from fat diet; Purina PicoLab Rodent Diet 20, W.F. Fisher and Son) or a high fat diet consisting of 60% kcal from fat (Purina TestDiet 58Y1, W.F. Fisher & Son) for 8–10 weeks^3^,^7^. All experiments on diet-induced obesity (DIO) mice were performed using mice that weighed at least 40 grams.

### Tacrolimus and iNOS inhibitor

The hindlimbs of control and experimental animals were treated bilaterally with depilatory cream (Nair, Church and Dwight Co., Princeton, NJ) and, beginning 24 hours later, were treated with topical tacrolimus (0.1% formulation with a dose of approximately 0.05 gm applied to the hindlimb twice daily; Astellas, Tokyo, Japan) or Vaseline cream (vehicle for tacrolimus) applied bilaterally twice a day for 14 days. In other animals, the potent and specific iNOS inhibitor, 1400W, (Cayman Chemical, Ann Arbor, Michigan) was dissolved in PBS (10 mg/mL) and administered intraperitoneally at a concentration of 10 mg/kg/day for 10 days^28^,^29^,^30^. Control animals were treated with PBS vehicle intraperitoneally for the same time period.

### Analysis of lymphatic function

Lymphoscintigraphy was performed as previously described to measure lymphatic uptake after distal hindlimb injection of technetium-99m (^99m^Tc)^3^. Briefly, 20 μL of ^99m^Tc was injected in the plantar side of the left distal hind paw and popliteal lymph node uptake was assessed for 90 minutes using an X-SPECT camera (Gamma Medica, Northridge, CA). Region-of-interest analysis was utilized to assess both peak and rate of popliteal lymph node using ASIPro software (CTI Molecular Imaging, Knoxville, TN). Experiments were performed using a minimum of 4 animals per group.

Lymphatic collecting vessel pumping was analyzed using near-infrared imaging and injection of indocyanine green (ICG)^53^,^54^. Briefly, ICG (a compound bound by albumin and taken up selectively by lymphatic vessels) was injected intradermally in the dorsal web space of the right distal hind-paw and then imaged 20 minutes later. Images of hindlimb lymphatic collectors were taken every eight seconds for 30 minutes using an EVOS EMCCD camera (Life Technologies, Carlsbad, CA), LED light source (CoolLED, Andover, UK) and a Zeiss V12 Stereolumar microscope (Caliper Life Sciences, Hopington, MA, USA). Animals were maintained under isofluorane anesthesia and placed on a heating pad to maintain a normal body temperature. The images were analyzed using region-of-interest analysis (Fiji software; NIH, Bethesda, MA) to quantify fluctuations in fluorescence intensity corresponding to lymphatic collecting vessel contraction^55^. Background noise was subtracted using digital imaging software (Fiji) and light intensity in the region of interest was analyzed. As lymphatic contraction capacity was noted to be augmented due to lymphatic stimulation while positioning the animal, the first 10 minutes of each 30 minute video were not analyzed to better study intrinsic lymphatic contraction. Lymphatic contraction frequency was quantified by measuring the total number of peaks in collecting vessel ICG signal and expressed as pulsations per minute.

Trafficking of peripherally injected dendritic cells (DCs) was performed using a modification of our previously published methods^3^. Briefly, CD45.1^+^ cells were harvested from spleens of B6.SJL-*Ptprc*^*a*^*Pepc*^*b*^/BoyJ (Jackson Laboratories, Bar Harbor, ME) using anti-CD11c^+^ magnetic microbead positive selection (Miltenyi Biotech, Gladbach, Germany). Isolated cells were resuspended and counted, and 1.5 × 10^6^ cells were injected into the plantar side of the left distal hind-paw. Eighteen hours after injection, the left popliteal lymph node was harvested, digested with a solution of Dispase II and Collagenase D (Roche Diagnostics, Mannheim, Germany) to prepare single cell suspensions, and analyzed using flow cytometry. Endogenous Fc receptor binding block was performed using anti-CD16/anti-CD32 and cells were stained using FITC-conjugated anti-CD45.1, PE-conjugated anti-CD11c and APC-conjugated MHC II antibodies (all from eBioscience, San Diego, CA). Quantification of migrating DCs (CD45.1^+^/CD11c^+^/MHC-II^*high*^) was determined using a LSRII flow cytometer (BD Biosciences, San Jose, CA) and FlowJo software (Tree Star, Ashland, OR). Cytometer settings were optimized and compensated for using C57BL/6NTac splenocytes obtained at the time of harvest. Each experiment was repeated in 4–5 animals per group.

In other experiments, we harvested full thickness skin from the hindlimb of experimental and control mice and created single cell suspensions using a Collagenase D, DNase I, and Dispase II enzyme digestion mix (all from Roche Diagnostics). Endogenous Fc receptor binding block was performed as outlined above and cells were stained APC/Cy7 anti-CD45, APC anti-CD3, PE/Cy7 anti-CD4, and PE anti-CD11b (all from eBioscience) to quantify inflammatory cell number and percentage. Quantification was performed using a LSRII flow cytometer and FlowJo software with cytometer optimization as outlined above. Each experiment was repeated in 4–5 animals per group.

### Histology

Cross-sectional histological sections were harvested from the left footpad and lower hind limbs, fixed using 4% paraformaldehyde (PFA; Affymetrix, Cleveland, OH), decalcified using EDTA (Sigma Aldrich, St. Louis, MO), and embedded in paraffin. In other experiments, cross sectional ear sections and longitudinal trachea sections were fixed in 4% PFA and embedded in optimal cutting temperature compound (OCT, Tissue Tek, Sakura, Torrance, CA). Immunohistochemical staining was performed according to our published methods^56^. Briefly, tissues were rehydrated followed by antigen unmasking using boiling sodium citrate (Sigma-Aldrich). Endogenous peroxidase activity was quenched using a 3% H_2_O_2_ solution and non-specific binding was blocked with 2% bovine serum albumin/10% secondary-antibody animal serum solution. Tissues were incubated in primary antibody overnight at 4 °C and in secondary antibodies conjugated with horseradish peroxidase for 1 hour. Staining was developed using diaminobenzamine complex (DAB, Dako, Carpinteria, CA) to obtain a brown stain or Deep Space Black (Biocare Medical, Concord, CA) to obtain a dark blue stain. Immunofluorescent staining was performed similarly, except that tissues were not incubated in 3% H_2_O_2_ or in secondary antibodies conjugated with horseradish peroxidase. Instead, immunofluorescent secondary antibodies (all from Life Technologies, Grand Island, NY) were used. Primary antibodies used were: anti-lymphatic vessel hyaluronan receptor (LYVE-1, R&D Systems, Minneapolis, MS), anti-iNOS (Abcam, Cambridge, MA), anti-CD45 (R&D Systems), anti-CD4 (R&D Systems), anti-CD3 (Dako), and anti-CD11b (R&D Systems). Slides were counter-stained with Mayer’s hematoxylin (Dako), dehydrated, and mounted using Mounting Medium (Thermo Scientific, Somerset, NJ) or Mowiol (Sigma-Aldrich).

Sections were analyzed using bright-field or fluorescent microscopy and scanned using a Mirax slide scanner (Zeiss, Munich, Germany). Perilymphatic inflammatory and iNOS^+^ cells were identified and counted in a minimum of 4–5 animals per group using 3–4 high powered fields (HPF) per animal by two blinded reviewers. All positively stained cells located within a 50μm radius of the most inflamed lymphatic vessel in randomly selected HPFs located in standardized quadrants of the limb were counted.

### Whole Mounts

Harvested ears were treated with a depilatory reagent (Nair), washed in a solution of phosphate buffered saline and Triton-X 100 (Sigma-Aldrich), followed by fixation in 4% PFA (Affymetrix) at 4 °C. The cartilage was then removed and the ear was split to expose the subcutaneous tissues. Sections were blocked in secondary serum and incubated in primary antibody solutions (anti-podoplanin (Abcam), anti-CD11b (R&D Systems), and anti-iNOS (BD Biosciences) overnight at 4 °C followed by fluorescent-conjugated secondary antibodies (Life Technologies).

### Statistical Analyses

All experiments were performed using a minimum of 4–6 animals/group unless otherwise noted. The student’s *t*-test was used to analyze differences between two groups, while ANOVA with post-hoc test were used to compare multiple groups. All data are presented as mean + standard deviation unless otherwise noted with p < 0.05 considered significant.

## Additional Information

**How to cite this article**: Torrisi, J. S. *et al.* Inhibition of Inflammation and iNOS Improves Lymphatic Function in Obesity. *Sci. Rep.* **6**, 19817; doi: 10.1038/srep19817 (2016).

## Supplementary Material

Supplementary Information

Supplemental Video 1

Supplemental Video 2

Supplemental Video 3

Supplemental Video 4

Supplemental Video 5

Supplemental Video 6

Supplemental Video 7

Supplemental Video 8
