# Supplementary material for: Inhibition of Inflammation and iNOS Improves Lymphatic Function in Obesity
Source: Sci Rep. 2016 Jan 22;6:19817. doi: 10.1038/srep19817 (PMC4726274; doi:10.1038/srep19817)
Supplement: Supplementary Information [file srep19817-s1.pdf]

Inhibition of Inflammation and iNOS Improves Lymphatic Function in Obesity

Jeremy S. Torrisi, BA, Geoffrey E. Hespe, BS, Daniel A. Cuzzone, MD, Ira L. Savetsky, MD, Matt D. Nitti, BA, Jason C. Gardenier, MD, Gabriela Garcia Nores, MD, Dawit Jowhar, PhD, Raghu P. Kataru, PhD, Babak J. Mehrara, MD FACS

Supplemental Figures

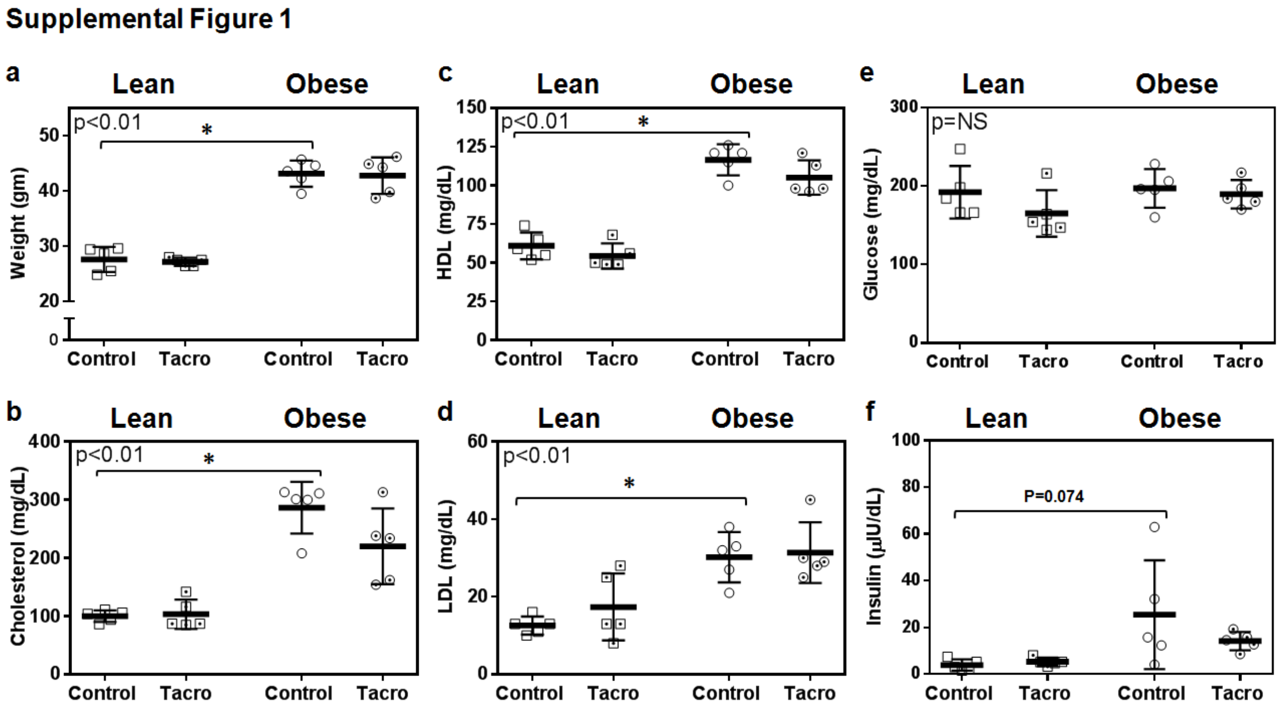

Supplemental Figure S1. Topical tacrolimus does not alter weight or metabolic parameters.

- **A.** Weights for experimental and control animals (n=5/group, p<0.01).
- **B.** Serum cholesterol levels of experimental and control mice (n=5/group, p<0.01).
- **C.** Serum HDL levels of experimental and control mice (n=5/group, p<0.01).
- **D.** Serum LDL levels of experimental and control mice (n=5/group, p<0.01).
- **E.** Serum glucose levels of experimental and control mice (n=5/group, p=NS).
- **F.** Serum insulin levels of experimental and control mice (n=5/group, p=0.074).

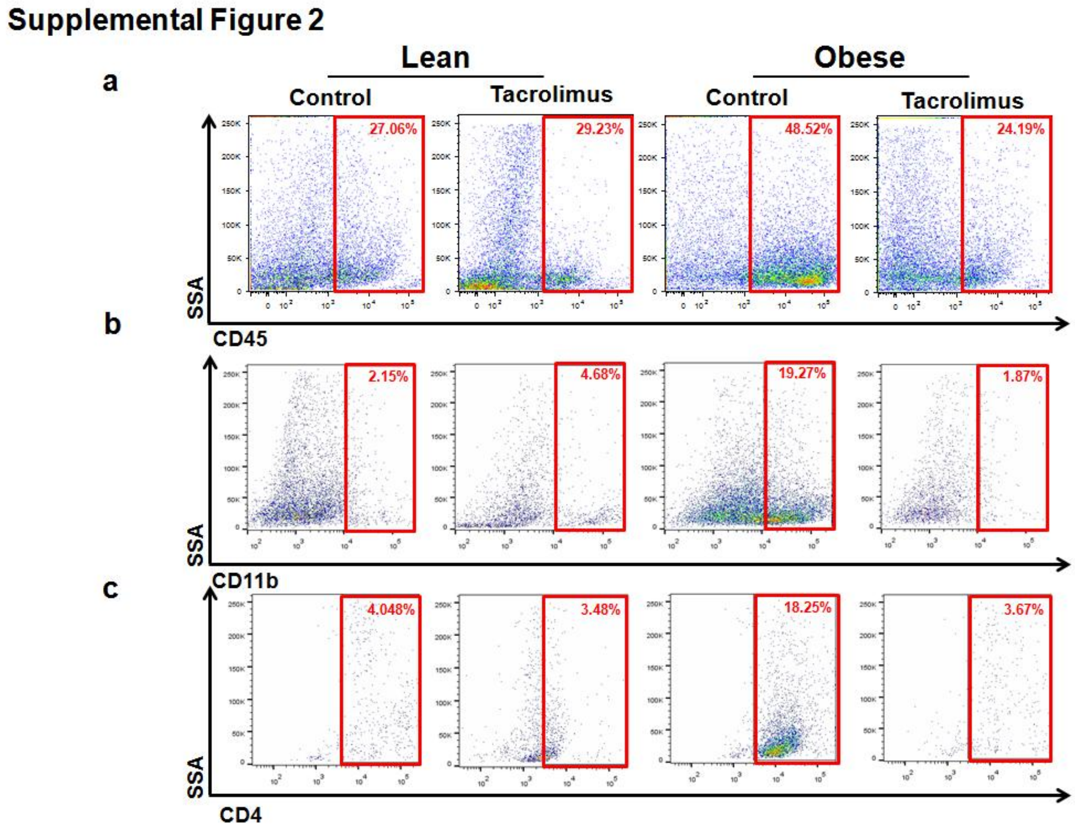

Supplemental Figure S2. Topical tacrolimus reduces local inflammation in obese animals.

A. Representative flow cytometry plots of hindlimb tissues to quantify $CD45^+$ cells (red box) as a percentage of live cells (n=4 animals/group).

B. Representative flow cytometry plots of hindlimb tissues to quantify macrophages ($CD11b^+/CD45^+$; red box) as a percentage of live cells (n=4 animals/group).

C. Representative flow cytometry plots of hindlimb tissues to quantify $CD4^+$ (red box) as a percentage of live cells (n=4 animals/group).

Supplemental Figure 3

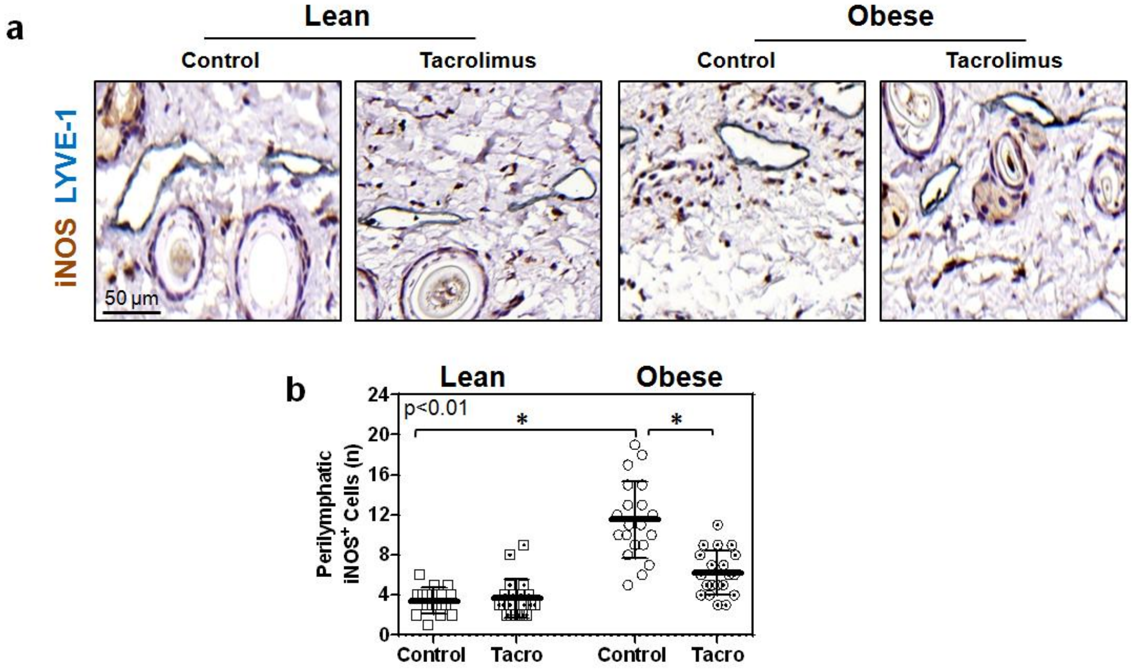

Supplemental Figure S3. Topical tacrolimus reduces perilymphatic iNOS$^+$ cell infiltration in hindlimbs.

A. Representative photomicrographs (40x) of hindlimb sections stained for iNOS and LYVE-1.

B. Quantification of perilymphatic iNOS$^+$ cells (n=5 animals per group with 4-5 HPF/animal, $p<0.01$).

Supplemental Figure 4

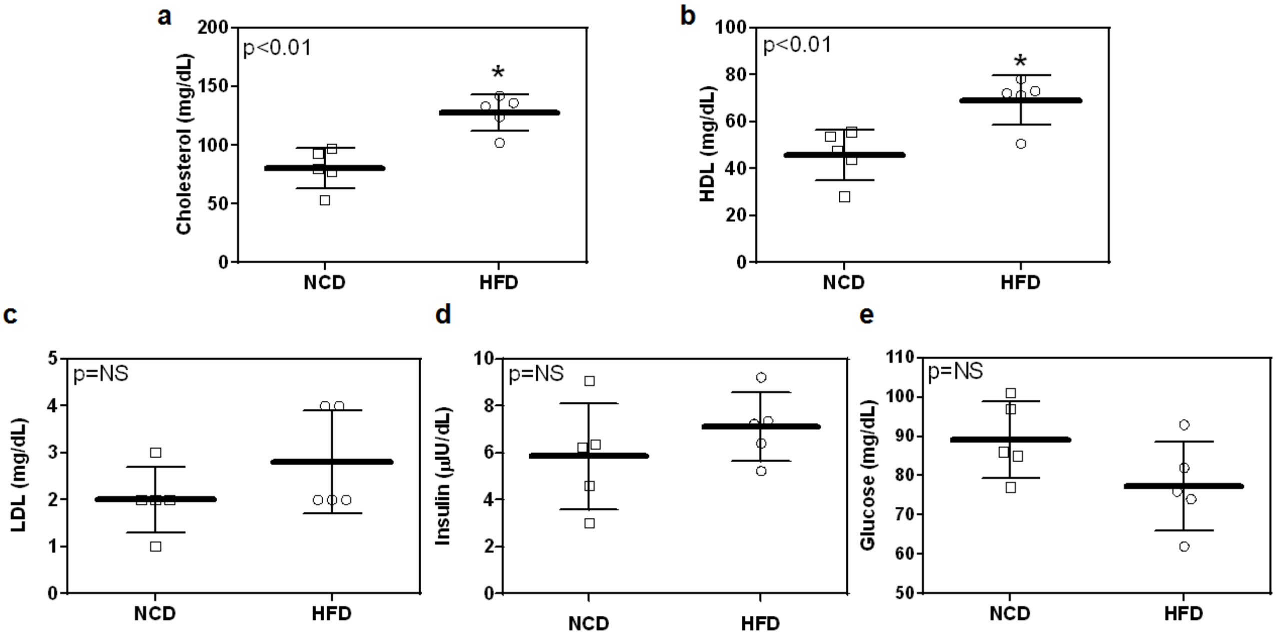

Supplemental Figure S4. CD4KO mice fed a high fat diet have mild changes in lipid profiles.

A. Serum cholesterol levels of NCD- and HFD-fed CD4KO mice (n=5/group, $p<0.01$).

B. Serum HDL levels of NCD- and HFD-fed CD4KO mice (n=5/group, $p<0.01$).

C. Serum LDL levels of NCD- and HFD-fed CD4KO mice (n=5/group, p=NS).

D. Serum insulin levels of NCD- and HFD-fed CD4KO mice (n=5/group, p=NS).

E. Serum glucose levels of NCD- and HFD-fed CD4KO mice (n=5/group, p=NS).

Supplemental Figure 5

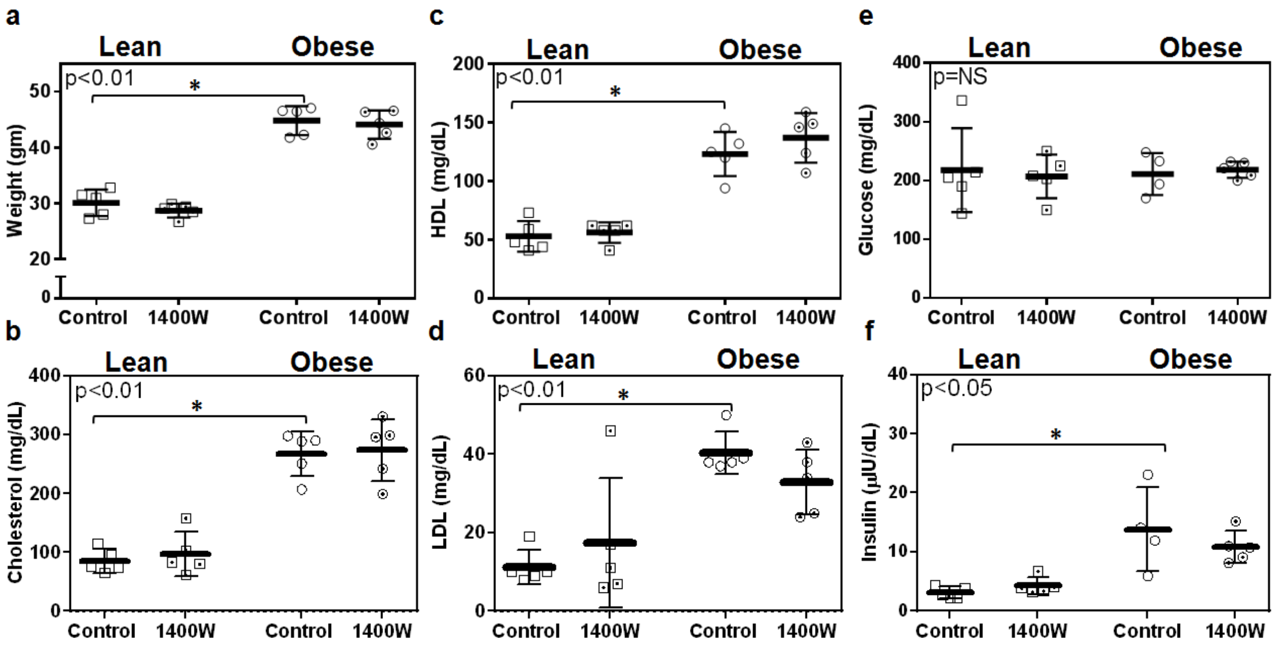

Supplemental Figure S5. Systemic iNOS inhibition does not alter weight or metabolic parameters.

- **A.** Weights for 1400W (iNOS inhibitor) treated and control animals (n=5 animals/group, $p<0.01$).
- **B.** Serum cholesterol levels for 1400W (iNOS inhibitor) treated and control mice (n=5 animals/group, $p<0.01$).
- **C.** Serum HDL levels for 1400W (iNOS inhibitor) treated and control mice (n=5 animals/group, $p<0.01$).
- **D.** Serum LDL levels for 1400W (iNOS inhibitor) treated and control mice (n=5 animals/group, $p<0.01$).
- **E.** Serum glucose levels for 1400W (iNOS inhibitor) treated and control mice (n=5 animals/group, p=NS).
- **F.** Serum insulin levels for 1400W (iNOS inhibitor) treated and control mice (n=5 animals/group, $p<0.05$).

Supplemental Figure 6

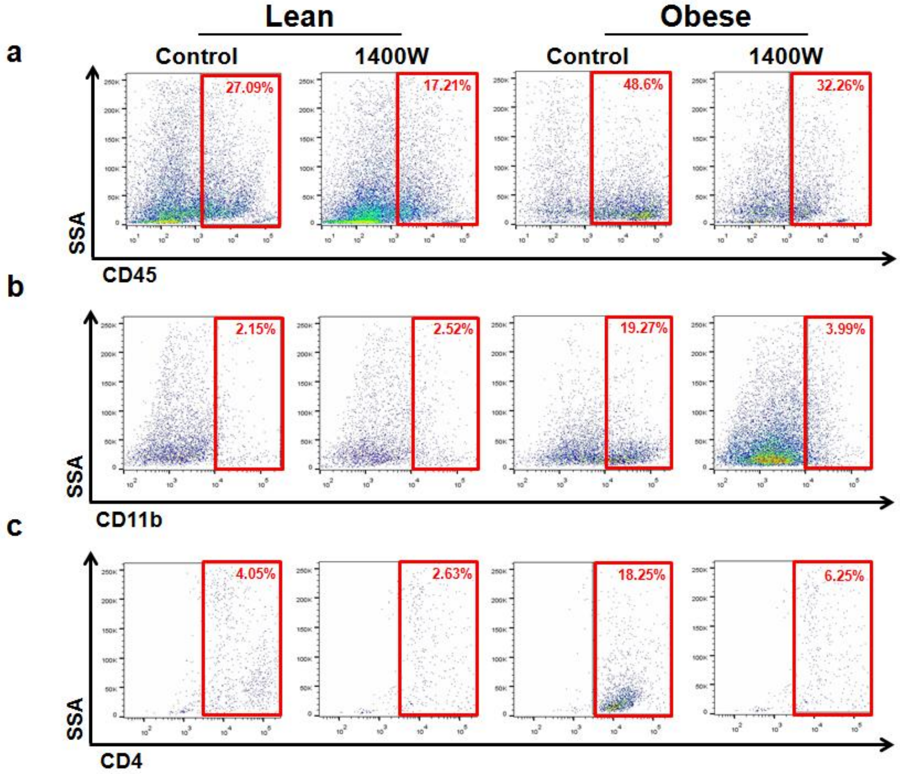

Supplemental Figure S6. iNOS inhibition reduces obesity-induced inflammation

A. Representative flow cytometry plots of hindlimb tissues to quantify $CD45^+$ cells (red box) as a percentage of live cells (n=4 animals/group).

B. Representative flow cytometry plots of hindlimb tissues to quantify macrophages ($CD11b^+$/$CD45^+$; red box) as a percentage of live cells (n=4 animals/group).

C. Representative flow cytometry plots of hindlimb tissues to quantify $CD4^+$ (red box) as a percentage of live cells (n=4 animals/group).

Supplemental Figure 7

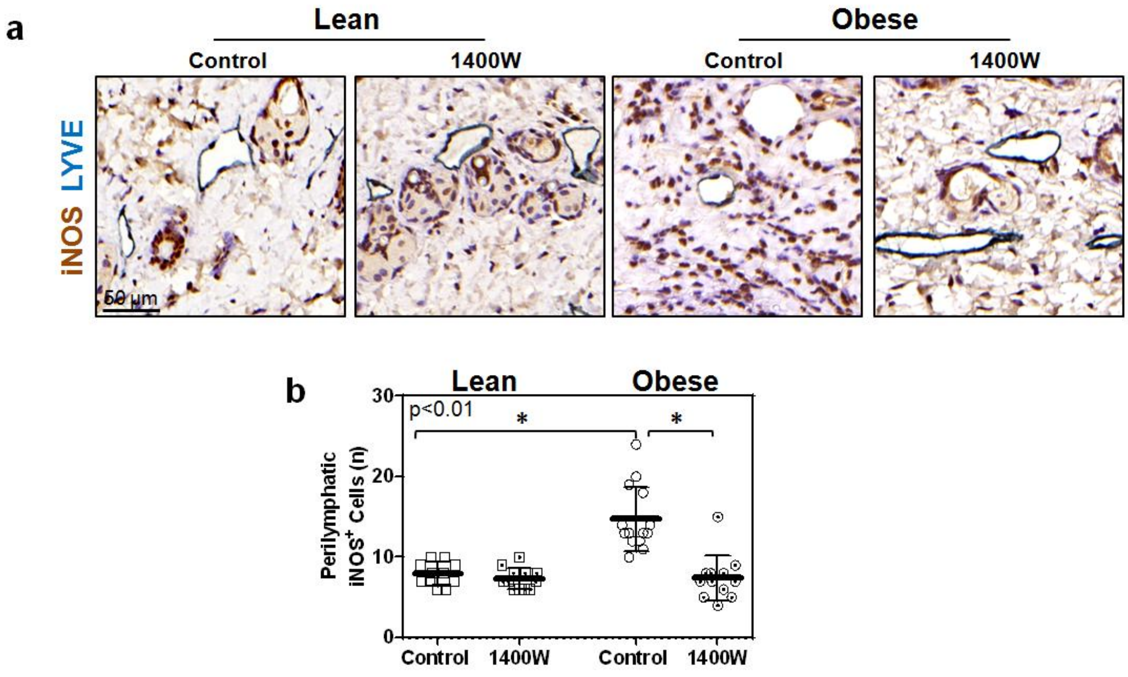

Supplemental Figure S7. iNOS inhibition reduces perilymphatic iNOS+ cell infiltration in hindlimbs

A. Representative photomicrographs (40x) of hindlimb sections stained for iNOS and LYVE-1.

B. Quantification of perilymphatic iNOS+ cells (n=5 animals per group with 4-5 HPF/animal, $p<0.01$).

Supplemental Videos

Supplemental Videos S1-4. Representative videos of lean control **(1)**, lean tacrolimus treated **(2)**, obese control **(3)**, and obese tacrolimus treated **(4)** animals analyzed using indocyanine green (ICG) lymphangiography. ICG was injected in the distal right hindlimb of the animal and the injection site is shown on the top right of the images. Videos are condensed into 30 second movies after 30 minutes of imaging. Quantification of lymphatic pulsations was performed during the final 20 minutes of the video.

Supplemental Videos S5-8. Representative videos of lean control **(1)**, lean 1400W treated **(2)**, obese control **(3)**, and obese 1400W treated **(4)** animals analyzed using indocyanine green (ICG) lymphangiography. ICG was injected in the distal right hindlimb of the animal and the injection site is shown on the top right of the images. Videos are condensed into 30 second movies after 30 minutes of imaging. Quantification of lymphatic pulsations was performed during the final 20 minutes of the video.
